# Supplementary material for: Content-rich biological network constructed by mining PubMed abstracts
Source: BMC Bioinformatics. 2004 Oct 8;5:147. doi: 10.1186/1471-2105-5-147 (PMC528731; doi:10.1186/1471-2105-5-147)
Supplement: Additional File 2 — The original results of the above study (non-essential files are deleted to keep the file size under the limit set by BMC bioinformatics). [file 1471-2105-5-147-S2.bz2 › chilibotAdditionalFile2/dip05/203ID7664335E693/html/ERCC6_CKN1.html]

 


 **ERCC6** and **CKN1** 
  
Found 57 abstracts in PubMed, retrieved 05.  
 

 What does Google say? 
 PDF only 
| .edu only 

---

**Interactive relationship** (e.g. stimulation, inhibition, etc)

**Non-interactive relationship** (e.g. studied together, co-existance, homology, etc.)

- Cockayne syndrome CS is a segmental premature aging syndrome in humans that has two complementation groups, CSA  [ **CKN1** ]  and CSB  [ **ERCC6** ] .  Ref: 12483520 Oncogene, 2002
- Two genes are involved CSA  [ **CKN1** ]  and CSB  [ **ERCC6** ] .  Ref: 12560492 Nucleic Acids Res, 2003
- The chondroitin sulfates CSA  [ **CKN1** ] , CSB  [ **ERCC6** ] , CSC had far less impact upon APTT with the FXa FXDP system.  Ref: 12430983 Can J Physiol Pharmacol, 2002
- The disease is caused by the disruption of CSA  [ **CKN1** ] , CSB  [ **ERCC6** ] , or some types of xeroderma pigmentosum genes.  Ref: 12665480 FASEB J, 2003
- ... survival, repair and recovery from transcription inhibition critically depend on CSA  [ **CKN1** ] , CSB  [ **ERCC6** ]  and UVS, the factors specific for transcription coupled NER.  Ref: 12531012 DNA Repair Amst , 2002
